# The Influence of Non-Starch Polysaccharide Enzymes in Diets Contaminated with Deoxynivalenol and Its Modified Form, Deoxynivalenol-3-Glucoside, on Growth Performance and Toxicokinetics in Broiler Chickens

**DOI:** 10.3390/toxins18080349

**Published:** 2026-08-17

**Authors:** Ellen van Eerden, Anna Arczewska-Wlosek, Zsofia Bata, Sylwester Swiatkiewicz, Regiane R. Santos

**Affiliations:** 1 Department of Research & Development, Schothorst Feed Research, Meerkoetenweg 26, 8218 NA Lelystad, The Netherlands; eveerden@schothorst.nl; 2 Department of Animal Nutrition and Feed Science, National Research Institute of Animal Production, Krakowska St. 1, 32-083 Balice, Polandsylwester.swiatkiewicz@iz.edu.pl (S.S.); 3 Research and Development Laboratory, Dr. Bata Ltd., Bajcsy-Zsilinnky utca 139, H-2364 Ócsa, Hungary

**Keywords:** DON, DON-3G, DON-3S, NSPase, toxicokinetics, excreta, poultry

## Abstract

The interaction between modified forms of deoxynivalenol (DON), such as DON-3-glucoside (DON-3G), and feed enzymes is underestimated. We assessed the interaction between DON and non-starch polysaccharide enzymes (NSPases) on growth performance, and excreta levels of DON and its metabolites (Experiment 1), as well as DON toxicokinetics (Experiment 2). Broiler chickens were fed maize-based diets naturally contaminated with low (LD; 516–792 μg DON/kg diet) or moderate (MD; 2395–3020 μg DON/kg diet) levels of DON. The LD and MD diets were supplemented with or without an NSPase blend. The MD diet impaired growth performance in broiler chickens up to 14 days of age, whereas no differences were observed in older birds. NSPase supplementation decreased body weight gain (BWG) in 28-day-old broiler chickens fed the MD diet and increased excreta levels of DON-3-sulfate (DON-3S). NSPase supplementation also improved the feed conversion ratio (FCR) without increasing BWG. Additionally, NSPase altered the toxicokinetic profile of DON in broiler chickens fed the MD diet, and the time to maximum plasma concentration (T_max_) of DON-3S was influenced by both NSPase supplementation and the DON level in the diet. To our knowledge, these findings provide the first evidence that NSPases influence DON toxicokinetics in poultry.

## 1. Introduction

Mycotoxins are unavoidable contaminants in animal feed. It has previously been reported that over 70% of feed and raw materials are contaminated with at least two mycotoxins [1]. Most studies, however, do not consider co-contamination with modified mycotoxins. Deoxynivalenol-3-glucoside (DON-3G) is a metabolite produced when a plant infected with *Fusarium* conjugates DON to glucose via enzymatic action (UDP-glycosyltransferases) as a defence mechanism against fungal infection; thus, DON-3G is a plant detoxification product of DON. This conjugation occurs on the plant surface or within the plant tissue, where the plant fibre and larger carbohydrates usually keep DON-3G bound [2]. This modified mycotoxin was first detected in maize and wheat samples 20 years ago [3] and has since been identified in various other cereal crops at variable levels, sometimes reaching 20% of the DON levels in the final feed [4].

After reaching the digestive tract of mammals, DON-3G undergoes non-specific hydrolysis by the host microbiota, generating free DON and thereby contributing to increased mycotoxin exposure [5]. In poultry, it has been suggested that DON-3G is not hydrolysed to DON [6]. However, to the best of our knowledge, the interactions of modified mycotoxins with poultry feed processing or added enzymes have not yet been studied, in contrast to food processing. For instance, bakery enzymes added to fermented dough resulted in a 145% increase in DON-3G, suggesting that α-amylases and xylanases could release this modified mycotoxin that was bound within the cereal [7]. Additionally, DON-3G levels increased by 48% during fermentation but did not remain stable after baking during rusk production, resulting in a transient increase in DON-3G [8]. Remarkably, DON-3G levels increased by 30–642% at lower temperatures (180 °C) and with a short baking time (10–15 min) compared with temperatures above 200 °C or baking times longer than 20 min [7]. An in vitro study demonstrated that cellulase cleaved up to 15% of DON-3G into DON, and cellobiase (a β-glucosidase) achieved cleavage of up to 73% [9].

Broiler diets are routinely supplemented with exogenous enzymes to increase nutrient availability, enhance digestibility, and reduce the viscosity of the intestinal contents. Diets with high viscosity can impair nutrient absorption, slow intestinal transit, and promote dysbiosis, especially in broiler chickens up to 28 days of age [10]. The increased intestinal viscosity results from soluble non-starch polysaccharides (NSPs) present in the diet, particularly when it contains high inclusion levels of wheat, barley, or rye [11,12,13]. To counteract this effect, diets are commonly supplemented with NSP enzyme products (NSPases), which are typically composed of xylanases and glucanases but may also be available as enzyme cocktails containing cellulase and β-glucosidase.

It was recently shown that overdosage of an NSPase cocktail (xylanase, β-glucanase, cellulase, pectinase, mannanase, galactanase, and arabinofuranosidase) had a positive effect on body weight gain (BWG) in broiler chickens fed a maize- or wheat-based diet from day 0 to 12, or a barley-based diet from day 12 to 23, whereas a negative impact on BWG and feed conversion ratio (FCR) was observed in broiler chickens fed a sorghum-based diet from day 23 to 35 [14]. The authors suggested that these inconsistencies were related to the variable NSP composition of the diets. Unfortunately, analysis of mycotoxin levels and their modified forms was beyond the scope of that study. In another study, broiler chickens were challenged with *Clostridium perfringens* to induce necrotic enteritis and fed either a maize- or wheat-based diet, both supplemented with xylanase and β-mannanase [15]. Again, the diet influenced NSPase activity, especially under challenge conditions, whereas supplementation with NSPase in the maize-based diet did not improve growth performance and even potentiated the negative impact of necrotic enteritis.

Based on the aforementioned studies, we hypothesised that dietary exposure to a mycotoxin such as DON may be underestimated and that the increased exposure to DON-3G, together with its enzymatic cleavage to DON, could affect the health and production performance of broiler chickens. Therefore, it is important to determine whether there is indeed a relationship between the presence of NSP enzymes and the increased DON plasma levels. Accordingly, the present study comprised two parallel experiments. In experiment 1, we aimed to evaluate the interaction between DON and its modified form, DON-3G, on growth performance and excreta levels of these mycotoxins. In experiment 2, we aimed to assess DON toxicokinetics in broiler chickens. In both experiments, the diets contained low (below the current European Union guidance value for DON in complete feed for broiler chickens, i.e., 1 mg/kg) or moderate levels of DON (2.3–3.0 mg/kg DON) with or without a cocktail of NSPases. In both experiments, the diets were produced using maize naturally contaminated with DON and DON-3G at either a low (LD) or moderate (MD) DON level.

## 2. Results

### 2.1. Experiment 1

#### 2.1.1. Growth Performance

The average body weight (BW) of the birds at the start of the trial was 43.3 g (42.6–44.3 g) across all treatments. At d14, BW was lower in broiler chickens fed the MD diet (520 g) than in those fed the LD diet (528 g). At d28, a significant DON × NSPase interaction was observed (*p* < 0.01), and the lowest BW was recorded in broiler chickens fed the LD diet without NSPase (1846 g) or the MD diet supplemented with NSPase (1850 g). No differences in BW were observed at d35 (Table 1).

During the starter period (d0–14) (Table 2), there was no interaction between DON level and NSPase with respect to growth performance. The lowest BWG at d14 was observed in broiler chickens fed the MD diet (474 g) compared with those fed the LD diet (485 g), whereas NSPase significantly decreased the FCR (1.142) compared with diets without NSPase (1.157).

In the grower period (d14–28) (Table 3), a significant interaction between DON level and NSPase was observed, resulting in significantly higher BWG in broiler chickens fed the LD diet supplemented with NSPase (1373 g) or the MD diet without NSPase (1375 g) than in those fed the LD diet without NSPase (1322 g) or the MD diet supplemented with NSPase (1333 g). Similarly, feed intake (FI) was significantly higher in broiler chickens fed the LD diet supplemented with NSPase (1828 g) or the MD diet without NSPase (1849 g) than in those fed the LD diet without NSPase (1781 g) or the MD diet supplemented with NSPase (1790 g). No differences in the FCR were observed.

Combining the starter and grower periods (d0–28) (Table 4), a significant interaction between DON level and NSPase was observed, resulting in increased BWG in broiler chickens fed the LD diet supplemented with NSPase (1862 g), whereas BWG was decreased when the MD diet was supplemented with NSPase (1801 g). Similarly, FI was increased in broiler chickens fed the LD diet supplemented with NSPase (2386 g), whereas it was decreased when broiler chickens were fed the MD diet supplemented with NSPase (2329 g). No differences in the FCR were observed.

In the finisher period (d28–35) (Table 5), a significant interaction between DON level and NSPase was observed for FCR. Supplementation of the MD diet with NSPase decreased the FCR (1.532) compared with the MD diet without NSPase (1.615), whereas no such effect was observed in the LD diet. Dietary supplementation with NSPase decreased FI (1301 vs. 1347 g). No other differences were observed.

Combining the complete feeding period (d0–35) (Table 6), a significant interaction between DON level and NSPase was observed. Broiler chickens fed the MD diet supplemented with NSPase had lower FI (3598 g) than those fed the other diets, resulting in a lower FCR (1.368). Overall mortality during the trial was low (2.5%) and was not affected by the treatments.

#### 2.1.2. Mycotoxins in Excreta

At d35, the highest DON-3-sulfate (DON-3S) peak area was observed in the excreta of broiler chickens fed the MD diet without NSPase (34,836), followed by the MD diet with NSPase (28,994), the LD diet with NSPase (12,019), and the LD diet without NSPase (6222). Broiler chickens fed the MD diet had significantly higher excretion of DON-3S than those fed the LD diet, regardless of the sampling time (d14, d28, or d35). Moreover, at d28, broiler chickens fed diets supplemented with NSPase had higher DON-3S peak areas (Table 7).

The highest peak areas of deepoxy-DON-3-sulfate (DOM-3S) were observed in the excreta of broiler chickens fed the MD diet compared with those fed the LD diet, regardless of NSPase supplementation or sampling time (d14, d28, or d35) (Table 8).

### 2.2. Experiment 2

DON was detected only in plasma samples from broiler chickens fed the MD diet, whereas dietary supplementation with NSPase did not affect plasma DON concentrations (Table 9). No DOM-1 was detected in the plasma samples.

**Figure 1:**
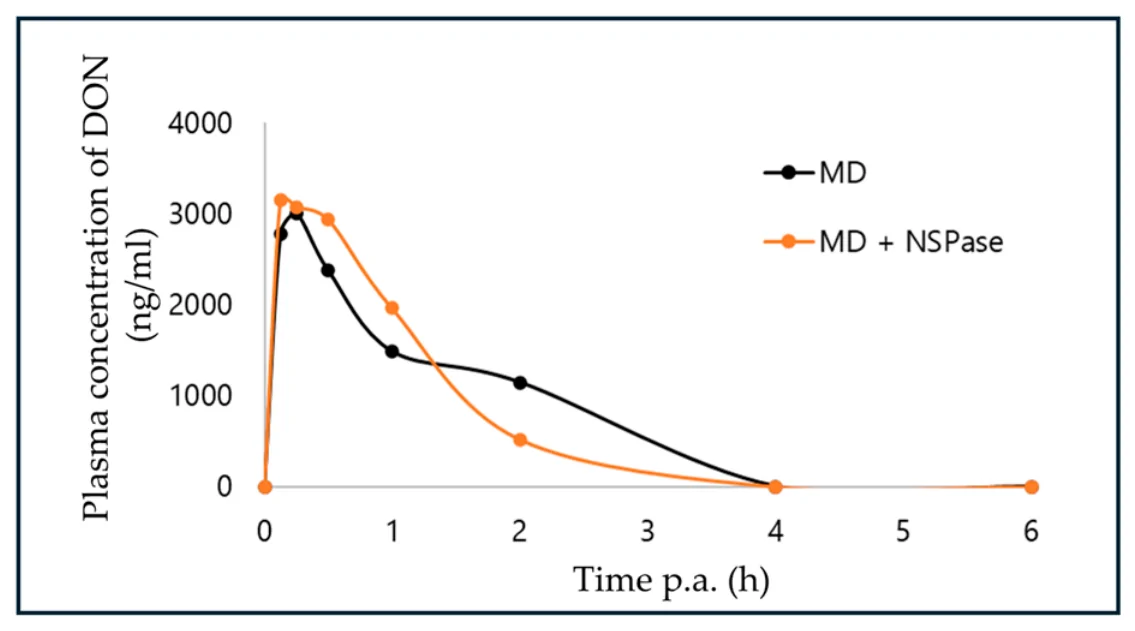
Mean plasma DON concentration–time curve following oral bolus administration of the MD diet with or without NSPase. p.a., post-administration.

Although no significant differences were observed in the toxicokinetic parameters, the shape of the plasma DON concentration–time curve differed when NSPase was added to the diet (Figure 1).

DON-3S was detected in all plasma samples. The area under the curve (AUC) and maximum plasma concentration (C_max_) of DON-3S were significantly higher in plasma from broiler chickens fed the MD diet than in those fed the LD diet. Supplementation of the LD diet with NSPase resulted in a time to maximum plasma concentration (T_max_) similar to that observed in broiler chickens fed the MD diet without NSPase. Conversely, supplementation of the MD diet with NSPase resulted in a T_max_ similar to that observed in broiler chickens fed the LD diet without NSPase (Table 10).

**Figure 2:**
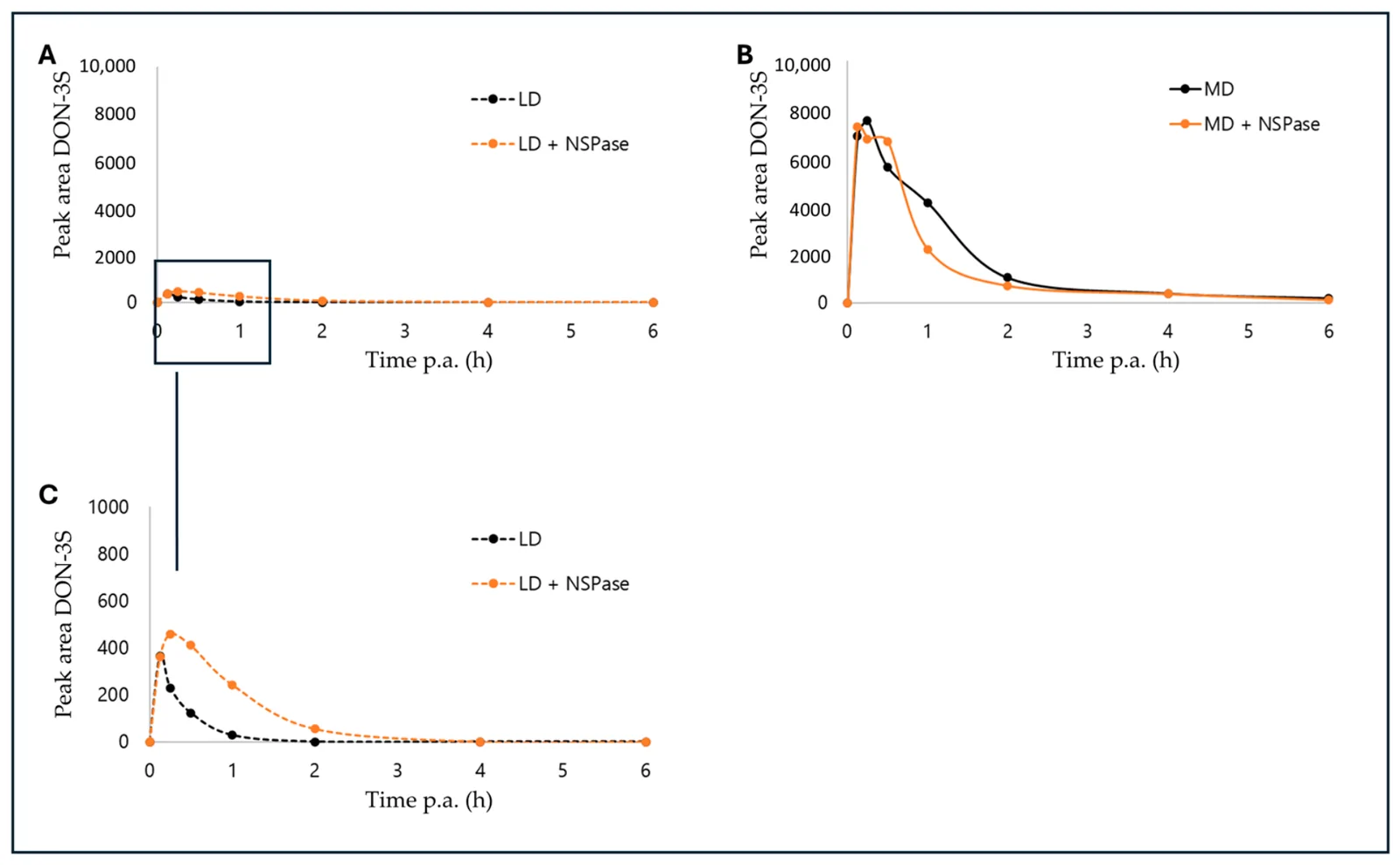
Mean DON-3S peak area following oral bolus administration of the (**A**) LD or (**B**) MD diet with or without NSPase. (**C**) Enlarged view of the DON-3S peak following administration of the LD diet with or without NSPase. p.a., post-administration.

Broiler chickens fed the MD diet supplemented with NSPase reached peak DON-3S concentrations earlier, whereas supplementation of the LD diet with NSPase increased T_max_ (Figure 2).

## 3. Discussion

The present study comprised two experiments to assess the interaction of an NSPase blend with DON and its modified form, DON-3G, with respect to growth performance and toxicokinetic parameters in broiler chickens. The variety of ingredients renders the formulation of mycotoxin-free diets impractical. For instance, in the present study, the diets included wheat (16.4–21.5%) and maize (47.5%). According to previous studies, DON contamination may be present in 34–100% and 32–100% of wheat and maize, respectively [16]. Hence, mycotoxins will be found in all diets under conditions that replicate farm reality. The LD group in the current study was contaminated with DON levels below the recently published European Union guidance value for DON in complete feed for fattening chickens, i.e., 1 mg/kg [17], whereas the MD group was contaminated with DON levels that impair broiler chicken performance [18].

In the first experiment, exposure to DON resulted in a significant decrease in BW and BWG in 14-day-old broiler chickens, whereas this effect was not observed in older birds. Previous studies demonstrated decreased BWG in 14-day-old broiler chickens fed diets naturally contaminated with 2.3 mg/kg DON [18] and decreased FI in 10-day-old broiler chickens fed diets artificially contaminated with DON (10 mg/kg) [19], suggesting that young broiler chickens are more sensitive to DON exposure than are older birds. On the other hand, dietary supplementation with NSPase improved FCR during the starter period (d0–14), resulting in improved FCR over the entire feeding period (d0–35). Poultry lack endogenous NSPases, and the absence of exogenous NSPases in the diet may result in suboptimal nutrient utilisation and an increased risk of digesta viscosity, depending on diet composition [20]. Considering the viscosity-enhancing properties of soluble NSPs, it should be noted that compared with wheat (14.6–21.7%) and barley (23.6–26.1%), maize contains a much lower concentration of soluble NSPs (4.2–11.8%) [21,22]. Maize was selected in the present study to avoid introducing increased intestinal viscosity as an additional factor. Although NSPases are not routinely added to maize-based diets, recent studies have shown promising effects because nutrients remain bound within the NSP fraction of maize, acting as a physical barrier during digestion [20]. Supplementation of broiler diets with xylanase in combination with β-glucanase or β-mannanase from 0 to 10 days of age enhanced growth performance and nutrient utilisation [20]. Growth performance of broiler chickens between 11 and 35 days of age was improved when xylanase and β-glucanase were added to the diet, but not when all three NSPases were combined. In the present trial, a DON × NSPase interaction was observed during the grower period, with BWG improving in broiler chickens fed the LD diet supplemented with NSPase, whereas the MD diet supplemented with NSPase resulted in decreased BWG. During the finisher period, no effect on BWG was observed, but FCR improved when the MD diet was supplemented with NSPase, whereas no such effect was observed with the LD diet.

In addition to performance parameters, excreta samples were collected to assess excreted mycotoxin levels. Only DON-3S and DOM-3S were detected in the excreta samples, whereas DON and DOM-1 were below the limit of detection. This finding agrees with previous observations, as DON is usually present at very low percentages in excreta (approximately 2%), whereas DOM-1 is rarely detected [23]. It has previously been demonstrated that DON-3S in excreta accounts for 89% of orally administered DON in chickens [24]. In the present trial, supplementation of the diets with NSPase increased DON-3S excretion at d28, probably indicating that more free DON was available in the diet. Although this measurement cannot be directly linked to increased or decreased intestinal absorption [25], the reduced BWG under MD conditions may indicate greater exposure of broiler chickens to DON when the NSPase blend was added to the diet. Poultry metabolise DON to DOM-3S, which is 2000 times less toxic than DON [23]. These authors proposed that the host microbiota convert DON into DOM-1, which is subsequently absorbed and sulphated to DOM-3S in the intestinal mucosa, liver, and/or kidney before being excreted, or alternatively, that DOM-3S results from retrograde movement of DON-3S followed by conversion to DOM-3S by the gut microbiota. In the present trial, the increased excreta levels of DON-3S were not accompanied by an increase in DOM-3S.

In Experiment 1, the birds had ad libitum access to feed. In Experiment 2, the birds received a bolus in a toxicokinetic study to investigate the effect of NSPase on the plasma concentrations of DON and its main metabolites; i.e., DON-3S, DOM-1, and DOM-3S. Although DOM-3S was detected in the excreta of broiler chickens after chronic dietary exposure, neither DOM-1 nor DOM-3S was detected in plasma during the toxicokinetic study. DOM-3S is formed by sulphation of DOM-1 in the intestine or by microbial conversion of DON-3S to DOM-3S [23], with the latter mechanism being the more plausible, as DON could not be converted to DOM-1 without a DON-decontaminating agent present in the diet. Unlike pigs [26], in which intestinal bacteria can partially convert DON to DOM-1, DON is rapidly absorbed in the upper intestine of poultry and converted to DON-3S in the intestinal mucosa, liver, and possibly the kidneys [23,27]. Consequently, only a small amount of DON reaches the caecum, where it could be converted to DOM-1 [28].

DON was detected only in the plasma of broiler chickens fed the MD diet, regardless of whether it was supplemented with NSPase. Although NSPase did not increase plasma DON concentrations in broiler chickens fed the MD diet, it altered the toxicokinetic profile. This alteration has implications when plasma DON concentrations are measured at a single time point, potentially leading to misinterpretation of the actual exposure depending on the sampling time. DON-3S was detected in all plasma samples, and supplementation of the LD diet with NSPase increased exposure to DON-3S, as indicated by increases in AUC and C_max_. These findings suggest that NSPase may increase the availability of DON by degrading the cell walls of the feedstuffs and releasing DON-3G, which can then be converted to DON. However, this effect was not observed with the MD diet, probably because NSPase activity had reached its capacity to degrade the available DON-3G to DON. Unfortunately, one of the limitations of the current study was that it was not possible to measure the disappearance of DON-3G in the biological samples. Therefore, the DON and DON-3S levels in plasma were considered indicative parameters of increased exposure to DON.

Importantly, dietary supplementation with NSPase also altered the toxicokinetic profile of DON-3S. Specifically, T_max_ increased when NSPase was added to the LD diet but decreased when it was added to the MD diet, indicating that the time to reach maximum plasma concentration depended on both NSPase supplementation and the DON level in the diet.

## 4. Conclusions

Based on the present experiments, exposure of young broiler chickens (14 days of age) to DON levels above 2000 μg/kg feed impairs growth performance, as reflected by reduced BWG. Furthermore, supplementation of a diet naturally contaminated with DON with NSPase for a prolonged period (28 days) impaired BWG, which may be associated with increased exposure of the birds to DON-3S, as measured in excreta. Based on the toxicokinetic study, NSPase may reach a maximum capacity to release free DON from the diet, as this increase was observed only in the LD diet. Moreover, NSPase improved FCR, even in a maize-based diet. Equally important, supplementation of a DON-contaminated diet with NSPase altered the toxicokinetic profile, which could potentially lead to misinterpretation of plasma DON concentrations when measured at a single time point. Despite these effects, supplementation of broiler diets with NSPases remains important for supporting optimal intestinal function and efficient nutrient utilisation. Therefore, the present results do not support removing NSP enzymes from diet formulations but rather highlight that the interaction between feed enzymes and mycotoxins should be considered, as their inclusion in contaminated diets may unintentionally increase the effective exposure of birds to DON. Finally, because the present study was performed using maize as the primary feedstuff (47.5% dietary inclusion), which contains much lower levels of soluble NSPs than wheat, caution should be exercised when extrapolating these results to wheat-based diets or other cereals with high NSP contents. Additional research using wheat as the source of DON and DON-3G, together with the assessment of DON-3G degradation, is still required.

## 5. Materials and Methods

### 5.1. Experiment 1

#### 5.1.1. Broiler Chickens and Housing

One-day-old male Ross 308 broiler chickens, purchased from a local commercial hatchery, were used in this study and randomly assigned to four dietary treatments of 210 chicks each (divided among 10 replicate pens with 21 chicks per pen). The birds were housed in 40 floor pens with wood shavings as bedding material at the broiler research facility of Schothorst Feed Research, Lelystad, The Netherlands. Each pen (2.2 m^2^) was equipped with one feeder and three drinking nipples. The birds were kept until 35 days of age. The ambient temperature gradually decreased from 34.5 °C upon arrival to 19.0 °C at 35 days of age. Room temperature and relative humidity were recorded daily. Continuous lighting was provided for the first 24 h to allow the birds to readily locate feed and water. Thereafter, the lighting schedule was 22L:2D for 1 day, followed by 8L:4D:10L:2D for the remainder of the experimental period, in compliance with EU legislation requiring a minimum of 6 h of darkness from the second day onwards, of which at least 4 h must be continuous. Birds were vaccinated against Newcastle disease at d10 and infectious bursal disease at d20. The health status of the flock was monitored by a poultry veterinarian. Throughout the experimental period, all birds were monitored daily for abnormalities, including abnormal behaviour, clinical signs of illness, and mortality.

#### 5.1.2. Diets and Experimental Design

The experiment comprised four dietary treatments in a 2 × 2 factorial design with DON at two levels (moderate [MD] and low [LD]) and diets supplemented with or without 1.1 g/kg of an NSP enzyme blend consisting of xylanases, glucanases, and cellulases.

The LD diet was prepared to achieve DON levels below the current EU guidance for the maximum DON levels in broiler chicken diets [17], and the MD diet was prepared to achieve DON levels able to impair intestinal integrity and growth performance, i.e., above 2.3 mg/kg [18].

Diets were prepared using maize batches naturally contaminated with different levels of DON, together with other mycotoxins. Treatments were randomly allocated to pens within blocks, with ten replicate pens per treatment. The pen was considered the experimental unit. The dietary treatments and their mycotoxin composition are summarised in Table 11. Although the supplemented diets were prepared from the same basal diet within each DON level (MD or LD), DON concentrations were analysed in all diets to calculate the mean contamination level. The mean concentrations of DON and DON-3G are presented in Table 11. All diets were analysed by an independent accredited laboratory (Primoris, Belgium). A multi-mycotoxin analysis was performed, with DON identified as the principal contaminant, whereas the other mycotoxins were present at negligible levels. The nutrient composition of the diets is provided in Table S1.

#### 5.1.3. Growth Performance

Broiler chickens were weighed by pen on d0, d14, d28, and d35. FI and mortality were recorded throughout the experimental period. BWG, FI, and FCR were calculated for the feeding phases d0–14, d14–28, and d28–35 and the overall experimental period (d0–35).

#### 5.1.4. Sampling and Analysis Methods

On d14 and d28, one broiler chicken per pen was randomly selected and euthanised, and blood samples were collected into heparinised tubes for plasma harvesting and subsequent metabolomic analysis. In addition, excreta samples were collected from each pen on d14, d28, and d35, freeze-dried, and stored until analysis for DON and its metabolites, as well as untargeted metabolomics.

Three hundred milligrams of homogenised faecal samples was transferred to a clean Falcon tube, and 4 mL of extraction solvent (MeOH:H_2_O:formic acid, 49.5:49.5:1; HPLC grade) was added. The slurry was shaken for 30 min, after which the solid material was pelleted by centrifugation at 6000 rpm for 1 min. The supernatant was collected, and the extraction was repeated twice with 3 mL of extraction solvent, with shaking for 20 and 10 min, respectively. The three extracts were combined, and 0.5 mL of the combined extract was diluted with 1 mL of MS-grade water. The mixture was centrifuged for 5 min at maximum speed (8000× *g*). The extract was filtered through a 0.22-μm HPLC filter, and 10 μL was injected into the HPLC-MS/MS system for analysis.

The excreta concentrations of DON, DOM-1, and DON-3G, together with the instrument responses for DON-3S and DOM-3S, were determined using HPLC-MS/MS. The limit of quantification (LOQ) was 7.1 ng/mL for DON, 5.2 ng/mL for DOM-1, and 1.3 ng/mL for DON-3G.

The analytical standard of DON was obtained from Fermentek (Jerusalem, Israel), and that of DOM-1 was obtained from LGC Standards (Wesel, Germany). All standards were stored according to the manufacturers’ recommendations. Water, methanol (MeOH), acetonitrile (ACN), glacial acetic acid, and formic acid for the mobile phases were of LC-MS grade and were obtained from Reanal Labor (Budapest, Hungary).

Chromatographic separation was achieved using a Kinetex C18 column (2.6 μm particle size, 4.6 mm internal diameter × 100 mm length; Phenomenex, Torrance, CA, USA) fitted with a guard column of the same type and maintained at 30 °C in a Shimadzu CTO-40C column oven (Shimadzu Corporation, Kyoto, Japan). Analyses were performed using a Shimadzu HPLC system (Shimadzu Corporation, Kyoto, Japan) coupled to a 6045 MS/MS detector. The mobile phases (MP) consisted of MeOH:H_2_O:AcOH (10:89:1) with 5 mM NH_4_Ac (MP A) and MeOH:H_2_O:AcOH (97:2:1) with 5 mM NH_4_Ac (MP B). A gradient elution programme with a constant flow rate of 0.45 mL/min was used for analyte separation: 0–1 min (100% MP A, 0% MP B), 1–2 min (linear gradient to 50% MP B), 2–4.5 min (linear gradient to 100% MP B), 4.5–6.0 min (100% MP B), 6.0–8.0 min (linear gradient to 100% MP A), and 8.0–11.5 min (100% MP A). The autosampler temperature was maintained at 20 °C. The LC column effluent was analysed using a Shimadzu 6045 mass spectrometer (Shimadzu Corporation, Kyoto, Japan) with the following settings: drying gas flow, 7 L/min; nebulising gas flow, 3 L/min; desolvation temperature, 444 °C; desolvation line temperature, 230 °C; and heating block temperature, 300 °C.

### 5.2. Experiment 2

#### Toxicokinetic Assay

Experiment 2 was conducted at the National Research Institute of Animal Production in Balice, Poland, and comprised four dietary treatments in a 2 × 2 factorial design with DON at two levels (moderate [MD] and low [LD]) and diets supplemented with or without 1.1 g/kg of an NSP enzyme blend consisting of xylanases, glucanases, and cellulases, as described for Experiment 1. Each treatment comprised five replicates, with each replicate consisting of one broiler chicken; thus, the experiment included 20 birds. The broiler chickens were obtained from a commercial hatchery and housed in floor pens (0.76 m^2^) with fresh wood shavings as bedding material. Feed and water were provided ad libitum. At 27 days of age, bolus administration and blood sampling were performed from five birds per experimental group. Broiler chickens were deprived of feed for 8 h before administration of a single bolus of the same grower diet used in Experiment 1. Briefly, a single bolus of grower diet (25 g/kg BW) was mixed with a 2% gelatine solution and administered by oral gavage. Blood samples (200 μL) were collected from each bird into heparinised tubes before bolus administration (t = 0) and at predetermined and fixed sampling intervals of 10, 20, 30, 60, 120, 240, and 360 min post-administration covering eight sampling time points per bird. Sampling was performed by trained personnel. Blood samples were centrifuged, and plasma was stored at −20 °C until analysis for DON and its metabolites (DON-3S, DOM-1, and DOM-3S.

Two hundred fifty microlitres of plasma was mixed with 750 μL of HPLC-grade ACN until protein precipitation occurred. The sample was centrifuged for 5 min at maximum speed (8000× *g*). The supernatant was transferred to a Wassermann tube and evaporated to dryness under a nitrogen stream at 40 °C. The residue was reconstituted in 200 μL of HPLC-MS-grade water, and 10 μL was injected into the HPLC-MS/MS system.

Samples were analysed by HPLC-MS/MS as described for Experiment 1. The LOQ was 4.7 ng/mL for DON and 6.7 ng/mL for DOM-1.

The toxicokinetic parameters were calculated using the concentrations of DON and DOM-1, together with the instrument responses for DON-3S and DOM-3S. The following parameters were calculated for each mycotoxin: area under the concentration–time curve from time zero to the last point above the LOQ (AUC_0→t_), C_max_, and T_max_.

### 5.3. Statistical Analysis

In Experiment 1, the pen was considered the experimental unit for all analyses, whereas in Experiment 2, the individual broiler chicken was considered the experimental unit. Data were analysed using analysis of variance (two-way analysis of variance with DON level and NSPase supplementation as the main effects) (GenStat version 23.0, 2023; Hemel Hempstead, UK). Treatment means were compared using the least significant difference test. Values of *p* ≤ 0.05 were considered statistically significant.

## Figures and Tables

**Table 1:** Average body weight of broiler chickens at d14, d28, and d35.

Treatment	DON	NSPase	d14		d28		d35	
LD	LD	No	524		1846	a	2700	
LD + NSPase	LD	Yes	532		1906	b	2718	
MD	MD	No	523		1898	b	2731	
MD + NSPase	MD	Yes	517		1850	a	2681	
	LD		528	b	1876		2709	
	MD		520	a	1874		2706	
		No	524		1872		2715	
		Yes	525		1878		2699	
Effect			*p*-value	LSD	*p*-value	LSD	*p*-value	LSD
DON × NSPase			0.07	10.6	<0.01	45.1	0.09	54.4
DON			0.03	7.5	0.90	31.9	0.87	38.5
NSPase			0.79	7.5	0.72	31.9	0.41	38.5

a,b Values within a column with different letters differ significantly (*p* < 0.05). LD: low DON level (516–792 μg/kg diet); MD: moderate DON level (2395–3020 μg/kg diet); LSD: least significant difference; DON: deoxynivalenol; NSPase: non-starch polysaccharide enzyme.

**Table 2:** Production performance of broiler chickens during the starter period (d0–14).

Treatment	DON	NSPase	BWG (g)		FI (g)		FCR (g/g)	
LD	LD	No	481		557		1.158	
LD + NSPase	LD	Yes	489		556		1.137	
MD	MD	No	474		548		1.157	
MD + NSPase	MD	Yes	474		544		1.147	
	LD		485	b	556		1.147	
	MD		474	a	546		1.152	
		No	477		552		1.157	b
		Yes	481		550		1.142	a
Effect			*p*-value	LSD	*p*-value	LSD	*p*-value	LSD
DON × NSPase			0.28	10.3	0.73	15.4	0.46	0.0209
DON			0.004	7.3	0.06	10.9	0.54	0.0148
NSPase			0.26	7.3	0.62	10.9	0.05	0.0148

a,b Values within a column with different letters differ significantly (*p* < 0.05). LD: low DON level (792 μg/kg diet); MD: moderate DON level (3020 μg/kg diet); LSD: least significant difference; BWG: body weight gain; FI: feed intake; FCR: feed conversion ratio; DON: deoxynivalenol; NSPase: non-starch polysaccharide enzyme.

**Table 3:** Production performance of broiler chickens during the grower period (d14–28).

Treatment	DON	NSPase	BWG		FI		FCR	
LD	LD	No	1322	a	1781	a	1.348	
LD + NSPase	LD	Yes	1373	b	1828	b	1.332	
MD	MD	No	1375	b	1849	b	1.345	
MD + NSPase	MD	Yes	1333	a	1790	a	1.343	
	LD		1347		1805		1.340	
	MD		1354		1819		1.344	
		No	1348		1815		1.346	
		Yes	1353		1809		1.338	
Effect			*p*-value	LSD	*p*-value	LSD	*p*-value	LSD
DON × NSPase			0.002	37.3	<0.001	34.9	0.32	0.0191
DON			0.63	26.4	0.23	24.7	0.49	0.0135
NSPase			0.72	26.4	0.63	24.7	0.19	0.0135

a,b Values within a column with different letters differ significantly (*p* < 0.05). LD: low DON level (635 μg/kg diet); MD: moderate DON level (2395 μg/kg diet); LSD: least significant difference; BWG: body weight gain; FI: feed intake; FCR: feed conversion ratio; DON: deoxynivalenol; NSPase: non-starch polysaccharide enzyme.

**Table 4:** Production performance of broiler chickens during the starter and grower periods (d0–28).

Treatment	DON	NSPase	BWG (g)		FI (g)		FCR (g/g)	
LD	LD	No	1803	a	2338	a	1.297	
LD + NSPase	LD	Yes	1862	b	2386	b	1.281	
MD	MD	No	1855	b	2404	b	1.296	
MD + NSPase	MD	Yes	1801	a	2329	a	1.293	
	LD		1832		2362		1.289	
	MD		1828		2366		1.295	
		No	1829		2371		1.297	
		Yes	1832		2357		1.287	
Effect			*p*-value	LSD	*p*-value	LSD	*p*-value	LSD
DON × NSPase			<0.01	45.3	<0.001	45.5	0.28	0.0162
DON			0.77	32.1	0.78	32.2	0.32	0.0115
NSPase			0.86	32.1	0.41	32.2	0.11	0.0115

a,b Values within a column with different letters differ significantly (*p* < 0.05). LD: low DON level (635–792 μg/kg diet); MD: moderate DON level (2395–3020 μg/kg diet); LSD: least significant difference; BWG: body weight gain; FI: feed intake; FCR: feed conversion ratio; DON: deoxynivalenol; NSPase: non-starch polysaccharide enzyme.

**Table 5:** Production performance of broiler chickens during the finisher period (d28–35).

Treatment	DON	NSPase	BWG (g)		FI (g)		FCR (g/g)	
LD	LD	No	854		1350		1.584	ab
LD + NSPase	LD	Yes	813		1329		1.637	b
MD	MD	No	833		1343		1.615	b
MD + NSPase	MD	Yes	831		1273		1.532	a
	LD		833		1340		1.611	
	MD		832		1308		1.574	
		No	843		1347	b	1.599	
		Yes	822		1301	a	1.585	
Effect			*p*-value	LSD	*p*-value	LSD	*p*-value	LSD
DON × NSPase			0.11	34.7	0.14	45.9	<0.01	0.0599
DON			0.93	24.6	0.06	32.5	0.09	0.0423
NSPase			0.09	24.6	<0.01	32.5	0.50	0.0423

a,b Values within a column with different letters differ significantly (*p* < 0.05). LD: low DON level (516 μg/kg diet); MD: moderate DON level (2490 μg/kg diet); LSD: least significant difference; BWG: body weight gain; FI: feed intake; FCR: feed conversion ratio; DON: deoxynivalenol; NSPase: non-starch polysaccharide enzyme.

**Table 6:** Production performance of broiler chickens during the complete feeding period (d0–35).

Treatment	DON	NSPase	BWG (g)		FI (g)		FCR (g/g)	
LD	LD	No	2657		3688	b	1.388	b
LD + NSPase	LD	Yes	2675		3715	b	1.389	b
MD	MD	No	2688		3747	b	1.394	b
MD + NSPase	MD	Yes	2630		3598	a	1.368	a
	LD		2666		3701		1.389	
	MD		2659		3672		1.381	
		No	2672		3717	b	1.391	b
		Yes	2653		3656	a	1.378	a
Effect			*p*-value	LSD	*p*-value	LSD	*p*-value	LSD
DON × NSPase			0.06	54.4	<0.01	78.2	0.03	0.0166
DON			0.73	38.5	0.29	55.3	0.20	0.0117
NSPase			0.31	38.5	0.04	55.3	0.04	0.0117

a,b Values within a column with different letters differ significantly (*p* < 0.05). LD: low DON level (516 μg/kg diet); MD: moderate DON level (2490 μg/kg diet); LSD: least significant difference; BWG: body weight gain; FI: feed intake; FCR: feed conversion ratio; DON: deoxynivalenol; NSPase: non-starch polysaccharide enzyme.

**Table 7:** Peak areas of DON-3S in the excreta of broiler chickens at d14, d28, and d35.

Treatment	DON	NSPase	d14		d28		d35	
LD	LD	No	5599		7574		6222	a
LD + NSPase	LD	Yes	8036		17,074		12,019	b
MD	MD	No	26,277		21,856		34,836	d
MD + NSPase	MD	Yes	24,723		32,563		28,994	c
	LD		6818	a	12,324	a	9121	
	MD		25,500	b	27,210	b	31,915	
		No	15,938		14,715	a	20,529	
		Yes	16,380		24,819	b	20,507	
Effect			*p*-value	LSD	*p*-value	LSD	*p*-value	LSD
DON × NSPase			0.14	3777	0.84	8334	<0.01	5041
DON			<0.001	2671	<0.001	5893	<0.001	3565
NSPase			0.74	2671	<0.01	5893	0.99	3565

a–d Values within a column with different letters differ significantly (*p* < 0.05). LD: low DON level (516–792 μg/kg diet); MD: moderate DON level (2395–3020 μg/kg diet); LSD: least significant difference; DON: deoxynivalenol; NSPase: non-starch polysaccharide enzyme.

**Table 8:** Peak areas of DOM-3S in the excreta of broiler chickens at d14, d28, and d35.

Treatment	DON	NSPase	d14		d28		d35	
LD	LD	No	7100		5982		7736	
LD + NSPase	LD	Yes	3384		13,278		5670	
MD	MD	No	27,486		21,636		27,312	
MD + NSPase	MD	Yes	24,224		25,266		32,963	
	LD		5242	a	9630	a	6703	a
	MD		25,855	b	23,451	b	30,137	b
		No	17,293		13,809		17,524	
		Yes	13,804		19,272		19,317	
Effect			*p*-value	LSD	*p*-value	LSD	*p*-value	LSD
DON × NSPase			0.95	8772	0.65	11,567	0.25	9493
DON			<0.001	6202	<0.01	8179	<0.001	6713
NSPase			0.26	6202	0.19	8179	0.59	6713

a,b Values within a column with different letters differ significantly (*p* < 0.05). LD: low DON level (516–792 μg/kg diet); MD: moderate DON level (2395–3020 μg/kg diet); LSD: least significant difference; DON: deoxynivalenol; NSPase: non-starch polysaccharide enzyme. No DON or deepoxy-DON (DOM-1) was detected in the excreta samples.

**Table 9:** Toxicokinetic characteristics of DON following a single oral bolus administration of the MD diet with or without NSPase.

Treatment	DON	NSPase	AUC (h.ng/mL)		C_max_ (ng/mL)		T_max_ (h)	
MD	MD	No	4.6		3.1		0.30	
MD + NSPase	MD	Yes	4.3		3.5		0.30	
			*p*-value	LSD	*p*-value	LSD	*p*-value	LSD
			0.72	0.60	0.15	0.17	1.00	0.05

MD: moderate DON level (2395 μg/kg diet); LSD: least significant difference; AUC: area under the curve; C_max_: maximum plasma concentration; T_max_: time to maximum plasma concentration; DON: deoxynivalenol; NSPase: non-starch polysaccharide enzyme. The experiment was carried out with five replicates, with each replicate consisting of one broiler chicken.

**Table 10:** Toxicokinetic characteristics of DON-3S following a single oral bolus administration of LD and MD diets with or without NSPase.

Treatment	DON	NSPase	AUC (h.ng/mL)		C_max_ (ng/mL)		T_max_ (h)	
LD	LD	No	160		368		0.20	a
LD + NSPase	LD	Yes	537		470		0.37	bc
MD	MD	No	11,624		7661		0.40	c
MD + NSPase	MD	Yes	8210		8200		0.24	ab
	LD		349	a	419	a	0.28	
	MD		9917	b	7931	b	0.32	
		No	5892		4014		0.30	
		Yes	4374		4335		0.30	
Effect			*p*-value	LSD	*p*-value	LSD	*p*-value	LSD
DON × NSPase			0.11	3356	0.47	904	<0.01	0.145
DON			<0.001	2373	<0.001	639	0.59	0.102
NSPase			0.19	2373	0.30	639	0.96	0.102

a–c Values within a column with different letters differ significantly (*p* < 0.05). LD: low DON level (635 μg/kg diet); MD: moderate DON level (2395 μg/kg diet); LSD: least significant difference; AUC: area under the curve; C_max_: maximal plasma concentration; T_max_: time to maximal plasma concentration; DON: deoxynivalenol; NSPase: non-starch polysaccharide enzyme. The experiment was carried out with five replicates, with each replicate consisting of one broiler chicken.

**Table 11:** Levels (μg/kg) of DON, DON-3G, and other mycotoxins in the starter (d0–14), grower (d14–28), and finisher (d28–35) diets.

	LD	MD
Starter (d0–14)		
DON	792.0	3020
3 + 15 ADON	36.3	87.1
DON-3G	107.0	293.5
Sum DON	935.3	3400
Zearalenone	144.6	282.0
FB_1_ + FB_2_	53.7	35.8
Nivalenol	54.8	-
Beauvericin	13.9	6.7
Enniatin B	3.6	-
Grower (d14–28)		
DON	635.0	2395
3 + 15 ADON	29.7	52.8
DON-3G	50.2	195.0
Sum DON	714.9	2643
Zearalenone	79.9	214.5
FB_1_ + FB_2_	27.1	59.2
Nivalenol	32.8	-
Beauvericin	22.8	26.0
Enniatin B	2.8	-
Finisher (d28–35)		
DON	516.0	2490
3 + 15 ADON	29.2	68.1
DON-3G	186.0	510.5
Sum DON	731.2	3069
Zearalenone	61.0	201.5
FB_1_ + FB_2_	38.9	64.4
T-2/HT-2	5.9	-
Beauvericin	19.8	20.0
Enniatin B	6.7	-

The analysed mycotoxins and their respective limits of quantification were aflatoxin B1 (1 µg/kg), aflatoxin B2 (1 µg/kg), aflatoxin G1 (1 µg/kg), aflatoxin G2 (1 µg/kg), alternariol (2 µg/kg), alternariol monomethyl ether (2 µg/kg), beauvericin (5 µg/kg), citrinin (10 µg/kg), cytochalasin E (2 µg/kg), deoxynivalenol (20 µg/kg), 3 + 15 acetyl-deoxynivalenol (3 + 15 ADON; 20 µg/kg), deoxynivalenol-3-glucoside (DON-3G; 20 µg/kg), diacetoxyscirpenol (5 µg/kg), enniatin A (5 µg/kg), enniatin A1 (5 µg/kg), enniatin B (5 µg/kg), enniatin B1 (5 µg/kg), fumonisins B1 + B2 (FB1 + FB2; 20 µg/kg), moniliformin (5 µg/kg), nivalenol (50 µg/kg), ochratoxin A (1 µg/kg), roquefortine C (5 µg/kg), sterigmatocystin (1 µg/kg), T-2/HT-2 toxin (10 µg/kg), and zearalenone (15 µg/kg). LD and LD + NSPase diets were prepared using the same marginally contaminated maize batch, whereas MD and MD + NSPase diets were prepared using the same contaminated maize batch.

## Data Availability

The original contributions presented in this study are included in the article/Supplementary Material. Further inquiries can be directed to the corresponding author.

## Supplementary Materials

The following supporting information can be downloaded at: https://www.mdpi.com/article/10.3390/toxins18080349/s1: Table S1: Composition of the experimental diets in the starter phase (d0-14), grower phase (d14-28), and finisher phase (d28-35) (values are expressed on and as-fed basis).

## Abbreviations

The following abbreviations are used in this manuscript:

3 + 15 ADON	3 + 15 Acetyl-deoxynivalenol
ACN	Acetonitrile
ANOVA	Analysis of variance
ARG	Arginine
AUC	Area under the curve
BW	Body weight
BWG	Body weight gain
Ca	Calcium
Cl	Chloride
C_max_	Maximal plasma concentration
DOM-1	Deepoxy-DON
DOM-3S	Deepoxy-DOM-3-sulfate
DON	Deoxynivalenol
DON-3G	Deoxynivalenol-3-glucoside
DON-3S	DON-3-sulfate
EFSA	European Food Safety Authority
FB_1_ + FB_2_	Fumonisins B_1_ + B_2_
FCR	Feed conversion ratio
FI	Feed intake
HPLC	High-performance liquid chromatography
ILE	Isoleucine
K	Potassium
LD	Low DON level
LOQ	Limit of quantification
LSD	Least significant difference
LYS	Lysine
M + C	Methionine + Cysteine
MD	Moderate DON level
MET	Methionine
MP	Mobile phase
MS	Mass spectrophotometry
Na	Sodium
NSPs	Non-starch polysaccharides
NSPase	NSP enzyme
P	Phosphorus
p.a.	Post-administration
SID	Standardized Ileal Digestible
THR	Threonine
T_max_	Time at maximal plasma concentration
TRP	Tryptophan
UDP-glycosyltransferases	Uridine Diphosphate-glycosiltransferases
VAL	Valine
ZEN	Zearalenone
