# Supplementary material for: The Influence of Non-Starch Polysaccharide Enzymes in Diets Contaminated with Deoxynivalenol and Its Modified Form, Deoxynivalenol-3-Glucoside, on Growth Performance and Toxicokinetics in Broiler Chickens
Source: Toxins (Basel). 2026 Aug 17;18(8):349. doi: 10.3390/toxins18080349 (PMC13517351; doi:10.3390/toxins18080349)
Supplement: Supplementary file 1 [file toxins-18-00349-s001.zip › toxins-4446440-supplementary.pdf]

Supplementary Materials: The Influence of Non-Starch Polysaccharide Enzymes in Diets Contaminated with Deoxynivalenol and Its Modified Form, Deoxynivalenol-3-Glucoside, on Growth Performance and Toxicokinetics in Broiler Chickens

Ellen van Eerden, Anna Arczewska-Wlosek, Zsofia Bata, Sylwester Swiatkiewicz and Regiane R. Santos

Table S1. Composition of the experimental diets in the starter phase (d0-14), grower phase (d14-28), and finisher phase (d28-35) (values are expressed on and as-fed basis).

**Ingredients (%)**	**Starter**	**Grower**	**Finisher**
Maize	47.5	47.5	47.5
Soybean meal	30.9	29.9	24.4
Wheat	16.4	16.5	21.5
Soybean oil	1.62	2.93	3.49
Salt	0.36	0.36	0.21
Limestone	0.88	0.81	0.92
Monocalcium Phosphate	1.22	0.87	0.72
Sodium Bicarbonate	0.00	0.00	0.22
Lysine HCl	0.27	0.24	0.22
DL-Methionine	0.27	0.25	0.19
Threonine	0.07	0.05	0.05
Valine	0.01	0.00	0.00
Choline chloride	0.07	0.07	0.07
Vitamin & Mineral premix	0.50	0.50	0.50
**Total**	**100.00**	**100.00**	**100.00**
**Nutrients (g/kg)**			
Apparent Metabolizable Energy (kcal/kg)	2900	3000	3075
Dry matter	888	888	888
Crude ash	55.11	50.35	45.99
Crude protein	209	204	183
Crude fat	46.62	59.46	64.81
Crude fibre	25.24	24.92	24.03
Starch	401	402	431
Ca	6.46	5.60	5.60
P	6.61	5.75	5.17
K	9.00	8.80	7.79
Na	1.50	1.50	1.50
Cl	3.29	3.23	2.31
SIDLYS	11.50	11.00	9.60
SIDMET	5.52	5.21	4.39
SIDM+C	8.17	7.81	6.82
SIDTHR	7.02	6.71	6.05
SIDTRP	2.08	2.03	1.79
SIDILE	7.65	7.47	6.59
SIDARG	12.19	11.89	10.37
SIDVAL	8.51	8.19	7.31
